# The mediating role of rumination between fear of disease progression and sleep disturbance in melanoma patients: a dual-perspective analysis based on person-centered and variable-centered approaches

**DOI:** 10.3389/fpubh.2025.1710386

**Published:** 2025-11-07

**Authors:** Qingjiang Huang, Fengwen Yue, Weitian Shi, Ying Lei, Ting Jiang

**Affiliations:** Burn Plastic and Aesthetic Surgery, Beijing Anzhen Nanchong Hospital of Capital Medical University & Nanchong Central Hospital, Nanchong, China

**Keywords:** melanoma patients, fear of disease progression, rumination, sleep disturbance, person-centered and variable-centered

## Abstract

**Background:**

Previous studies have confirmed that melanoma patients often experience intense fear of disease progression, with a significantly higher incidence of sleep disorders compared to the general population. However, the relationship between these two factors and the underlying psychological mechanisms remain unclear. To address the gaps in previous research, the present study employs a dual-methodological approach, incorporating both person-centered and variable-centered strategies, to comprehensively examine the association and heterogeneity between fear of disease progression and sleep disturbances among melanoma patients.

**Methods:**

A cross-sectional survey design was implemented, utilizing convenience sampling to enlist a total of 501 melanoma patients from three tertiary-level hospitals located in Sichuan Province, China. Data collection took place between May and July 2025.

**Results:**

Findings from the variable-centered analysis indicated that fear of disease progression not only exerted a direct positive association with the occurrence of sleep disturbances but also mediated this relationship through the amplifying effect of rumination. The person-centered analysis delineated three distinct subgroups of patients. Among these, the subgroup characterized by medium fear of disease progression – high rumination demonstrated markedly higher levels of sleep disturbance in comparison to the other two groups. This suggests that individuals who experience both medium fear of disease progression and high rumination are particularly susceptible to severe sleep-related issues.

**Conclusion:**

The results of this study underscore the critical role of rumination as a key psychological mechanism that mediates the impact of fear of disease progression on sleep quality among melanoma patients. From a clinical and psychological intervention standpoint, implementing cognitive-behavioral strategies aimed at reducing rumination within this high-risk subgroup may serve as an effective approach to mitigate fear levels and enhance overall sleep quality.

## 1 Introduction

Melanoma is a highly metastatic and dangerous malignancy resulting from the malignant transformation of melanocytes, which are responsible for pigment production (1). According to statistics from the World Health Organization, the global incidence of newly diagnosed melanoma cases exceeded 320,000 in 2020. Notably, in advanced-stage patients, the five-year survival rate is only between 20 and 30% (2). With the ongoing changes in environmental factors and lifestyle, the incidence of melanoma has shown a significant upward trend, particularly among urban populations (3). Previous research has primarily focused on treatment methods for melanoma, aiming to alleviate the physiological pain experienced by patients (4, 5). However, the psychological burdens faced by melanoma patients have not received adequate attention in prior studies. Research has indicated that cancer often triggers a range of psychological issues in patients, including anxiety, depression, and post-traumatic stress disorder, which can adversely affect treatment adherence and quality of life (6, 7). Among these, the fear of disease progression refers to the continuous worry patients have about tumor recurrence, metastasis, or deterioration (8, 9). This concern arises not only from the unpredictable nature of the disease itself but also from the ambiguity of medical information and the individual coping strategies of patients (10, 11). In the melanoma patient population, given the disease’s highly invasive nature and elevated risk of recurrence, the incidence of fear of disease progression can be as high as 50–70%, a figure significantly greater than that observed in other types of cancer (12). However, previous studies have only focused on the impact of fear of disease progression on cancer patients’ quality of life (13), death anxiety (14), and perceived stigma (15). Sleep disturbances, which are common complications among cancer patients (16), have yet to be thoroughly analyzed in relation to the fear of disease progression. According to the findings of Savard and Morin (17), the prevalence of sleep disturbances among cancer patients is approximately 30–50%. This not only exacerbates patients’ fatigue and cognitive decline but may also promote tumor progression through immune suppression mechanisms. Therefore, this study analyzes the relationship between the fear of disease progression and sleep disturbances in melanoma patients, as well as the underlying psychological mechanisms, which holds significant clinical importance.

Sleep disturbances in cancer patients are primarily characterized by difficulty falling asleep, frequent awakenings during the night, and early waking (18, 19). In melanoma patients, chemotherapy and targeted therapies exhibit neurotoxicity, making sleep issues particularly pronounced (20, 21). Chronic insomnia can upregulate inflammatory factors, thereby promoting the remodeling of the tumor microenvironment (22). Existing studies mostly attribute sleep disturbances to factors such as pain, medication side effects, and hospital environments (23, 24); however, the role of psychological factors has not been sufficiently emphasized. Specifically, the fear of disease progression, as a chronic stressor, can activate the sympathetic nervous system, leading to disruptions in sleep architecture (25). According to the Predisposing, Precipitating, and Perpetuating factors model (26), the diagnosis and treatment of melanoma can serve as precipitating factors that activate pre-existing anxiety tendencies in patients, while intrusive thoughts and avoidance behaviors related to the fear of disease progression become perpetuating factors. Specifically, the fear of disease progression prompts patients to exhibit heightened cognitive vigilance, which manifests as an excessive interpretation of bodily sensations, such as misinterpreting itching or pain as signs of tumor metastasis (9, 27). This state of heightened vigilance can transform into cognitive arousal before sleep, inhibiting the autonomic nervous system’s calming necessary for sleep onset (28). Additionally, melanoma patients’ repetitive skin lesion examinations may delay sleep onset and shorten sleep duration (29). Thus, this study aims to: (1) verify whether the fear of disease progression in melanoma patients has a positive impact on sleep disturbances; and (2) further analyze the internal mechanisms and existing heterogeneity between the two.

Rumination is a cognitive process characterized by the repetitive contemplation of negative events and emotions, often leading to the amplification of emotions and a decrease in problem-solving abilities (30). While both fear of disease progression and rumination are interrelated cognitive-emotional reactions frequently observed in oncology populations, they operate at distinct levels within the psychological processing hierarchy. According to the Cognitive Appraisal Theory (31), the fear of disease progression primarily represents an immediate threat appraisal—a primary cognitive-affective evaluation of the disease as a potential source of harm, encompassing vigilance, worry, and catastrophic anticipation regarding tumor recurrence or deterioration. In contrast, rumination corresponds to a secondary metacognitive process (32), which persistently revisits and magnifies these initial appraisals through repetitive and self-focused thought loops. This distinction implies that fear of disease progression captures “what patients fear,” whereas rumination reflects “how patients think about their fear.” Recognizing this conceptual boundary provides a clearer theoretical rationale for testing rumination as a mediating mechanism in the current study, thereby enhancing the construct validity of our proposed model. In the field of tumor psychology, rumination has been confirmed as an important mechanism affecting the psychological adaptation of cancer patients (33, 34). Recently, convergent evidence has emerged from the broader cancer population. Amani et al. (35) demonstrated that rumination significantly mediated the association between fear of recurrence, cancer-related fatigue, psychological distress, and insomnia among cancer survivors. According to the Response Styles Theory (32), rumination exacerbates and prolongs negative emotional states, hinders patients’ problem-solving abilities, and reinforces catastrophic thinking about the disease. Specifically, on one hand, rumination can maintain and amplify negative emotions, extending the patients’ experience of suffering and depleting cognitive resources, thereby weakening their ability to effectively cope with stress (36); on the other hand, rumination enhances patients’ attentional bias toward threatening information and memory retrieval, forming cognitive distortions that lead patients to catastrophically interpret neutral or ambiguous situations (37). Therefore, rumination can predict higher levels of anxiety, depression, and fear of disease progression (38), and it can lead to physiological dysfunctions such as insomnia and decreased sleep quality by disrupting cognitive-emotional regulation and exacerbating cognitive arousal before sleep (39, 40). Based on the Attention Control Theory, rumination consumes patients’ cognitive resources, making it difficult for them to detach from negative thoughts, thus prolonging sleep latency (41). However, this prolonged difficulty in falling asleep further reinforces patients’ fear of disease progression (42). Therefore, high levels of fear of disease progression trigger patients’ repetitive negative thoughts about disease threats, manifesting as persistent immersion in negative treatment information, heightening the intensity of fear emotions, and ultimately leading to exacerbated sleep disturbances.

Previous research has separately focused on the fear of disease progression and sleep disturbances in cancer patients, but the connection between the two has not been extensively discussed, and there is a scarcity of studies analyzing the internal mechanisms of both. Currently, variable-centered approaches dominate the existing literature, assuming patient homogeneity but neglecting the patient heterogeneity resulting from individual differences.

The integration of variable-centered and person-centered approaches in this study is grounded in the complementary nature of these two methodological paradigms. The variable-centered approach, rooted in classic statistical modeling traditions, aims to examine the overall associations among constructs and estimate average effects across the population. This perspective assumes population homogeneity and is effective for identifying generalizable pathways, such as the mediating role of cognitive processes (43). In contrast, the person-centered approach—such as latent profile analysis (LPA)—focuses on identifying subpopulations that share specific configurations of psychological characteristics (44). This perspective acknowledges heterogeneity and allows researchers to detect how different combinations of fear of disease progression and rumination patterns may manifest distinct psychological risk profiles.

The theoretical novelty of the current study therefore lies in bridging these two perspectives within a single analytical framework. By combining variable-centered mediation analysis and person-centered profiling, this research not only validates the directional associations among variables but also captures the within-group diversity and pattern-level complexity that traditional models neglect. This dual-perspective approach aligns with multi-level conceptualizations in psycho-oncology, where both common pathways (e.g., cognitive mediation) and individual heterogeneity (e.g., coping typologies) are essential for precision psychological interventions. Thus, this methodological integration provides both a macro-level understanding of the mechanism linking fear of progression, rumination, and sleep disturbance, and a micro-level identification of vulnerable subgroups requiring tailored clinical strategies.

Based on the theoretical rationale above, three hypotheses are proposed:

*H1*: Fear of disease progression is positively associated with sleep disturbance among melanoma patients.

*H2*: Rumination mediates the relationship between fear of disease progression and sleep disturbance.

*H3*: Distinct latent subgroups of fear of disease progression–rumination combinations differ significantly in the level of sleep disturbance.

## 2 Materials and methods

### 2.1 Participants

#### 2.1.1 Study design

This study utilized a cross-sectional survey design, employing convenience sampling, conducted from May to July 2025 in the dermatology and oncology departments of three tertiary hospitals in Sichuan Province, China. The cross-sectional design is suitable for assessing the associations and potential mechanisms between variables at a specific point in time, particularly for preliminarily exploring the mediating pathways among psychological variables and group heterogeneity. Therefore, this study combines both variable-centered and person-centered perspectives. On one hand, it examines the mediating role of rumination between fear of disease progression and sleep disturbances; on the other hand, it identifies the latent categories of fear of disease progression and rumination. By comparing the differences in sleep disturbances across categories, it reveals the heterogeneous patterns within the group.

#### 2.1.2 Ethical considerations

This study strictly adheres to the ethical guidelines of the Declaration of Helsinki. The research protocol has been approved by the Ethics Review Committee of Nanchong Central Hospital (No.: 2025116). All patients were thoroughly informed about the study’s purpose, processes, and potential risks before participating and signed a written informed consent form. The questionnaire was completed anonymously, and the data were used solely for scientific research, with access limited to the research team members. All participants had the right to withdraw from the survey at any time without affecting their medical rights.

#### 2.1.3 Minimum sample size estimation

To ensure statistical power, we used G*Power 3.1 software to estimate the minimum sample size. Assuming a significance level of *α* = 0.05, a power (1 – *β*) of 0.95, and an effect size of *f*^2^ = 0.15, with a hypothesized number of 12 predictor variables in the model (including demographic variables, fear of disease progression, rumination, and sleep disturbances), the minimum sample size calculated was 184 cases. Additionally, latent profile analysis typically requires at least 50 samples per category, and the total sample size must meet the model fitting requirements. Considering the need for 5–6 latent category analyses in latent profile analysis and to prevent invalid questionnaires and dropouts, the final target sample size was set at 500 cases.

#### 2.1.4 Participant recruitment process

The recruitment process employed convenience sampling to minimize selection bias. During their scheduled outpatient visits, potential participants were initially identified by their attending physicians based on preliminary medical records. Subsequently, researchers provided them with a detailed explanation of the study’s purpose, procedures, potential risks, and benefits. Interested participants received a written information sheet and were given at least 24 h to consider participation. Written informed consent was obtained from all participants before any data collection. Patients were then required to complete either an electronic or paper questionnaire.

#### 2.1.5 Inclusion and exclusion criteria

Inclusion Criteria: (1) Patients diagnosed with cutaneous melanoma confirmed by histopathological examination (45); (2) Ability to understand and complete self-report questionnaires in Chinese; (3) Voluntary participation in the study and signing of a written informed consent form; (4) Patients at any treatment stage after diagnosis; (5) Patients with normal cognitive abilities and language communication skills.

Exclusion Criteria: (1) Significant cognitive impairment or severe mental disorders recorded in medical history or determined by the treating physician; (2) Patients with another active malignancy; (3) Patients with severe visual or auditory impairments that hinder their ability to complete the assessment; (4) Melanoma patients diagnosed with sleep disorders and receiving treatment, to reduce the assessment of sleep disturbances related to cancer experiences; (5) Participants who have engaged in similar studies in the past week.

#### 2.1.6 Study sample

A total of 534 melanoma patients were recruited for this study. Among them, 16 participants had two or more comorbidities; 6 patients were currently undergoing treatment for sleep disturbances; and 3 patients had significant cognitive impairments. During the data cleaning phase, 3 incomplete questionnaires were filtered out, and 5 questionnaires with strong regularity in responses were excluded. Therefore, the effective sample size for this study was 501 questionnaires, resulting in a response rate of 93.82%. Of these, 296 were male patients (59.10%) and 205 were female patients (49.90%). The mean age of patients was 31.67 ± 9.363 years. Patients with education ranging from middle school to high school numbered 246 (49.10%), married patients totaled 260 (51.90%), and working patients accounted for 253 (50.50%). Detailed demographic information is presented in Table 1.

**Table 1:** Demographic information of all the participants.

Variable	Items	Number	Percentage
Gender	Male	296	59.10%
	Female	205	40.90%
Educational background	Primary school and below	27	5.40%
	From junior high school to senior high school	246	49.10%
	Bachelor’s degree or above	228	45.50%
Marital status	Divorced	98	19.60%
	Widowed	16	3.20%
	Unmarried	127	25.30%
	Married	260	51.90%
Working	Worker	253	50.50%
	Unemployment	97	19.40%
	Retirement	7	1.40%
	Student	27	5.40%
	Liberal professions	117	23.40%
Raise a child	No.	240	47.90%
	Yes	261	52.10%
Place of residence	City	315	62.90%
	Rural	186	37.10%
Monthly income level	≤1,000￥	33	6.60%
	1,001–3,000￥	84	16.80%
	3,001–5,000￥	197	39.30%
	5,001–8,000￥	160	31.90%
	≥8,001￥	27	5.40%
Cancer staging	0 stage	150	29.90%
	I stage	155	30.90%
	II stage	142	28.30%
	III stage	44	8.80%
	IV stage	10	2.00%
Primary site of cancer	Trunk	121	24.20%
	Limbs	171	34.10%
	Head and neck	55	11.00%
	Soles/fingertips	73	14.60%
	Other	81	16.20%
Sleep environment	Sleep alone	130	25.90%
	Living with a partner	203	40.50%
	Living with children/family	141	28.10%
	Hospitalization	27	5.40%
Smoking	No	266	53.10%
	Yes	235	46.90%
Drinking alcohol	No	267	53.30%
	Yes	234	46.70%

### 2.2 Research instruments

#### 2.2.1 Fear of Disease Progression Scale

The measurement of fear of disease progression among melanoma patients is derived from the Fear of Disease Progression Scale for chronic illnesses developed by Herschbach et al. (46). This scale consists of a total of 43 items across five dimensions: Affective Reactions (13 items), Partnership/Family (7 items), Occupation (7 items), Loss of Autonomy (7 items), and Coping with Anxiety (9 items). The scale has been widely applied in cancer populations (8) and has been validated for cultural adaptability in Chinese populations (47). In this study, a 5-point Likert scale was used for scoring (1 = strongly disagree, 5 = strongly agree), with higher scores indicating greater fear of melanoma. Confirmatory factor analysis was conducted using AMOS 29.0 software, and the results indicated good model fit for the scale. Reliability analysis revealed excellent internal consistency, as shown in Table 2.

**Table 2:** Confirmatory factor analysis and reliability analysis of the study variables.

Variable	*χ*^2^/df	CFI	TLI	GFI	RMSEA	Cronbach’s α
Fear of disease progression	1.767	0.907	0.902	0.864	0.039	0.950
Rumination	1.693	0.936	0.932	0.908	0.037	0.937
Sleep disorders	2.010	0.908	0.898	0.910	0.045	0.880

#### 2.2.2 Sleep Disturbance Scale

The measurement items for sleep disturbances in this study are derived from the Sleep Disturbance Scale developed by Buysse et al. (48), which assesses patients’ sleep depth, quality, and restorative aspects. Previous studies on Chinese populations have shown that this scale has good reliability and cultural adaptability (49, 50). The scale consists of 27 items, primarily measuring sleep quality, difficulty falling asleep, nighttime awakenings, and sleep satisfaction. A 5-point Likert scale was used for scoring, where 1 indicates strong disagreement and 5 indicates strong agreement, with higher scores reflecting more severe sleep disturbances. Confirmatory factor analysis demonstrated good model fit for the scale, and reliability analysis showed good internal consistency, as indicated in Table 2.

#### 2.2.3 Rumination Scale

The measurement items for rumination are based on the Disease Rumination Scale developed by Soo and Sherman (51), which includes a total of 32 items across four dimensions: Intrusiveness (11 items), Instrumentality (8 items), Meaning-Seeking (4 items), and Reflection (9 items). This scale aims to assess individuals’ rumination in the context of physical illness, such as in breast cancer patients (51), adult cancer patients (52), and migraine patients (53). Additionally, Zhang et al. (54) adapted this scale into Chinese, validating its cultural adaptability and reliability in the Chinese patient population. A 5-point Likert scale was used for scoring (1 = strongly disagree, 5 = strongly agree), with higher scores indicating more severe rumination. Confirmatory factor analysis showed good model fit for the scale, and reliability analysis revealed excellent internal consistency, as shown in Table 2.

### 2.3 Statistical analysis

First, we utilized AMOS 29.0 software to construct a structural equation model for the variables and conducted confirmatory factor analysis to assess the model fit of all measurement variables. Next, we employed SPSS 27.0 software to analyze the reliability of each variable and performed analyses for common method bias, descriptive statistics, and correlation analysis. Third, we analyzed the mediating effect of rumination using Model 4 of the Process macro in SPSS. The core of Process is based on regression analysis, but it automates the complex model specification, coefficient calculation, and statistical testing processes. Fourth, we constructed latent profiles of fear of disease progression and rumination using Mplus 8 software, selecting the optimal latent profile based on AIC, BIC, aBIC, and Entropy indices. Finally, we conducted *post hoc* analyses to examine the differences in sleep disturbances across the optimal latent profiles of fear of disease progression and rumination.

## 3 Results

### 3.1 Common method bias

To further reduce the issue of common method bias arising from self-reported measures, we informed patients that all questionnaire responses would be treated with strict confidentiality and anonymity to alleviate social desirability pressure. During the questionnaire design phase, we also balanced the order of items to avoid logical cues.

Finally, we included all measurement items in exploratory factor analysis and conducted Harman’s single-factor test. The results indicated that the maximum explained variance of the first extracted factor was 21.732%, which is below the critical threshold of 40%. Therefore, there is no common method bias in this study.

### 3.2 Descriptive statistics and correlation analysis

#### 3.2.1 Evidence for discriminant validity

To examine whether fear of disease progression and rumination are empirically distinct constructs, we conducted analyses of discriminant validity based on the Fornell–Larcker criterion and the heterotrait–monotrait ratio of correlations (HTMT). The square roots of the average variance extracted (AVE) values for fear of disease progression (√AVE = 0.78) and rumination (√AVE = 0.81) exceeded their inter-construct correlation (*r* = 0.456), satisfying the Fornell–Larcker criterion for discriminant validity. Additionally, the HTMT ratio between FoP and rumination was 0.54, well below the conservative threshold of 0.85 (55). These results suggest that while fear of disease progression and rumination are moderately related, they measure conceptually distinct psychological processes, thereby ruling out problematic multicollinearity (VIF ≤ 2.31 for all predictors).

#### 3.2.2 Descriptive statistics and correlation analysis

We conducted descriptive statistics and correlation analysis for fear of disease progression, sleep disturbances, and rumination, as shown in Table 3. The study found that fear of disease progression, rumination, and sleep disturbances ranged from 3.339 to 3.384, all exceeding the midpoint value of 2.5, indicating that melanoma patients experience high levels of fear regarding disease progression, rumination, and sleep disturbances. The skewness of the core variables ranged from −0.647 to −0.111, which is less than |3|, and the kurtosis ranged from 2.602 to 0.640, which is less than |10|. According to Kline (56), the core variables in this study conform to an approximately normal distribution.

**Table 3:** Descriptive statistics and correlation analysis results of fear of disease progression, rumination and sleep disorders.

Variable	*M*	SD	Skewness	Kurtosis	1	2	3
Fear of disease progression	3.339	0.615	−0.111	0.640	1		
Rumination	3.384	0.632	−0.647	2.141	0.456***	1	
Sleep disorders	3.347	0.532	−0.412	2.602	0.434***	0.681***	1

**p < 0.001.

Based on the criteria proposed by Cohen (57), the absolute values of the correlation coefficients (*r*) are interpreted as follows: |*r*| < 0.300 indicates a weak correlation; 0.300 ≤ |*r*| < 0.500 indicates a moderate correlation; and |*r*| ≥ 0.500 indicates a strong correlation. A strong positive correlation was found between rumination and sleep disturbances (*r* = 0.681, *p* < 0.001), indicating that the more severe the rumination tendencies in melanoma patients, the more severe their sleep disturbances. There were moderate positive correlations between fear of disease progression and rumination (*r* = 0.456, *p* < 0.001) and between fear of disease progression and sleep disturbances (*r* = 0.434, *p* < 0.001). This suggests that the stronger the fear of disease progression, the more likely melanoma patients are to experience more frequent rumination and more severe sleep disturbances.

### 3.3 Mediation analysis

One-way ANOVA revealed that participants’ demographic information, including education background, marital status, occupation, children, residence, income, cancer stage, primary site, smoking, and drinking, significantly affected sleep disturbances, rumination, or fear of disease progression (*p* < 0.05). Therefore, we included these variables as control variables in the model, with rumination as a mediator, fear of disease progression as the independent variable, and sleep disturbances as the dependent variable, using Process Model 4 to test the mediating effect of rumination (Bootstrap sample: 5000).

The study found that fear of disease progression was positively associated with rumination (*β* = 0.429, *p* < 0.001, 95% CI = [0.345, 0.513]). Fear of disease progression also showed a significant positive association with sleep disturbances (*β* = 0.118, *p* < 0.001, 95% CI = [0.055, 0.181]). Similarly, rumination was positively related to sleep disturbances (*β* = 0.517, *p* < 0.001, 95% CI = [0.456, 0.577]), as shown in Table 4.

**Table 4:** Mediating effect path coefficients of rumination.

Regression equation		Overall fit index			Significance of regression coefficient		
Outcome variables	Predictive variables	*R*	*R* ^2^	*F*	*β*	*t*	95%CI
Rumination	Fear of disease progression	0.497	0.247	14.557***	0.429	10.014***	[0.345, 0.513]
Sleep disturbances	Fear of disease progression	0.713	0.508	42.003***	0.118	3.682***	[0.055, 0.181]
	Rumination				0.517	16.765***	[0.456, 0.577]

***p < 0.001.

It should be noted that, given the cross-sectional nature of the data, these statistical associations represent concurrent relationships rather than verified causal effects. The mediation model reflects a theoretical formulation consistent with cognitive–affective frameworks, serving an explanatory rather than confirmatory purpose.

**Figure 1:**
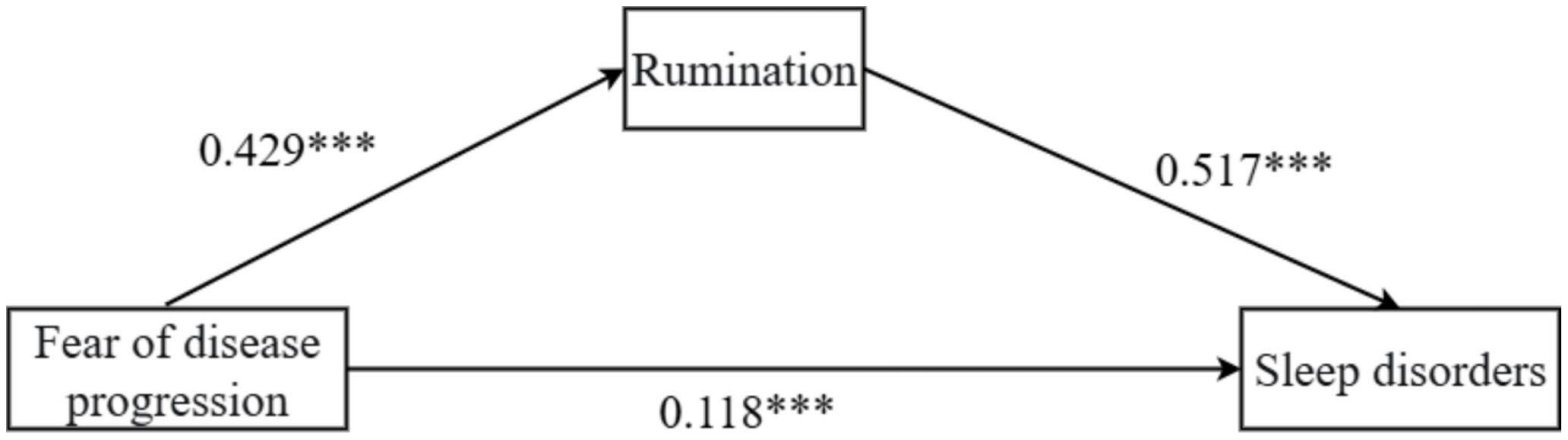
Path coefficients of the intermediate analysis in rumination, ***p < 0.001.

Further analysis of the mediation effect of rumination revealed that the total effect of fear of disease progression on sleep disturbances in melanoma patients was 0.339, with 65.2% of the effect being mediated by rumination, as shown in Table 5 and Figure 1. Fear of disease progression had a significant direct effect on sleep disturbances (*β* = 0.118, SE = 0.032, 95% CI = [0.055, 0.181]). At the same time, the mediating effect of rumination between fear of disease progression and sleep disturbances was significant (*β* = 0.211, SE = 0.061, 95% CI = [0.117, 0.356]).

**Table 5:** Decomposition effect of the mediating effect of rumination.

Type of effect	*β*	SE	LLCI	ULCI	Percentage
Total effect	0.339	0.037	0.267	0.411	
Direct effect	0.118	0.032	0.055	0.181	34.8%
Indirect effect	0.221	0.061	0.117	0.356	65.2%

### 3.4 Latent profile analysis

Next, we used Mplus 8 software to construct a latent profile model for the dimensions of fear of disease progression and sleep disturbances in melanoma patients. We developed a total of 1 to 5 latent profile models and assessed and selected the optimal fitting model. The model fit indices included AIC, BIC, aBIC, LMR, BLRT, and Entropy. AIC (Akaike Information Criterion), BIC (Bayesian Information Criterion), and aBIC (Sample-Size Adjusted BIC) values are lower for models comparing k profiles to k-1 profiles, indicating greater accuracy of the profile model. A *p*-value of LMR (Lo–Mendell–Rubin Likelihood Ratio Test) and BLRT (Bootstrapped Likelihood Ratio Test) less than 0.05 indicates that the K-class model is superior to the K-1 model. Entropy measures the precision and certainty of profile classification, with higher Entropy indicating smaller classification errors. The better the distinction between profiles, the more effective the classification.

As shown in Table 6, as the number of latent profile models increased, the AIC, BIC, and aBIC values continuously decreased, indicating that the classification of latent profile models became more accurate. However, compared to the 2-class latent profile model (Entropy = 0.876), the 3-class latent profile model had a higher entropy value (Entropy = 0.934), the interpretive stability of the smallest subgroup (1.4%) should be viewed with caution, given its limited sample size and potential parameter instability. The *p*-value of LMR for the 3-class latent profile model was lower (*p* = 0.002) compared to the 4-class latent profile model (*p* = 0.102). Therefore, this study selected the 3-class latent profile model as the main model for its accuracy and clarity.

**Table 6:** Fitted indices for latent profiles of fear disease process and rumination.

Profile	AIC	BIC	aBIC	Entropy	LMR (p)	BLRT (p)	Smallest proportion per class
1	9616.413	9692.312	9635.179	–	–	–	–
2	8195.924	8313.989	8225.115	0.876	0.024	<0.001	0.615/0.385
3	7490.428	7650.659	7530.044	0.934	0.002	<0.001	0.625/0.014/0.361
4	6761.293	6963.690	6811.335	0.936	0.102	<0.001	0.573/0.014/0.225/0.158
5	6223.833	6468.396	6284.300	0.955	0.106	<0.001	0.135/0.014/0.014/0.574/0.263

**Figure 2:**
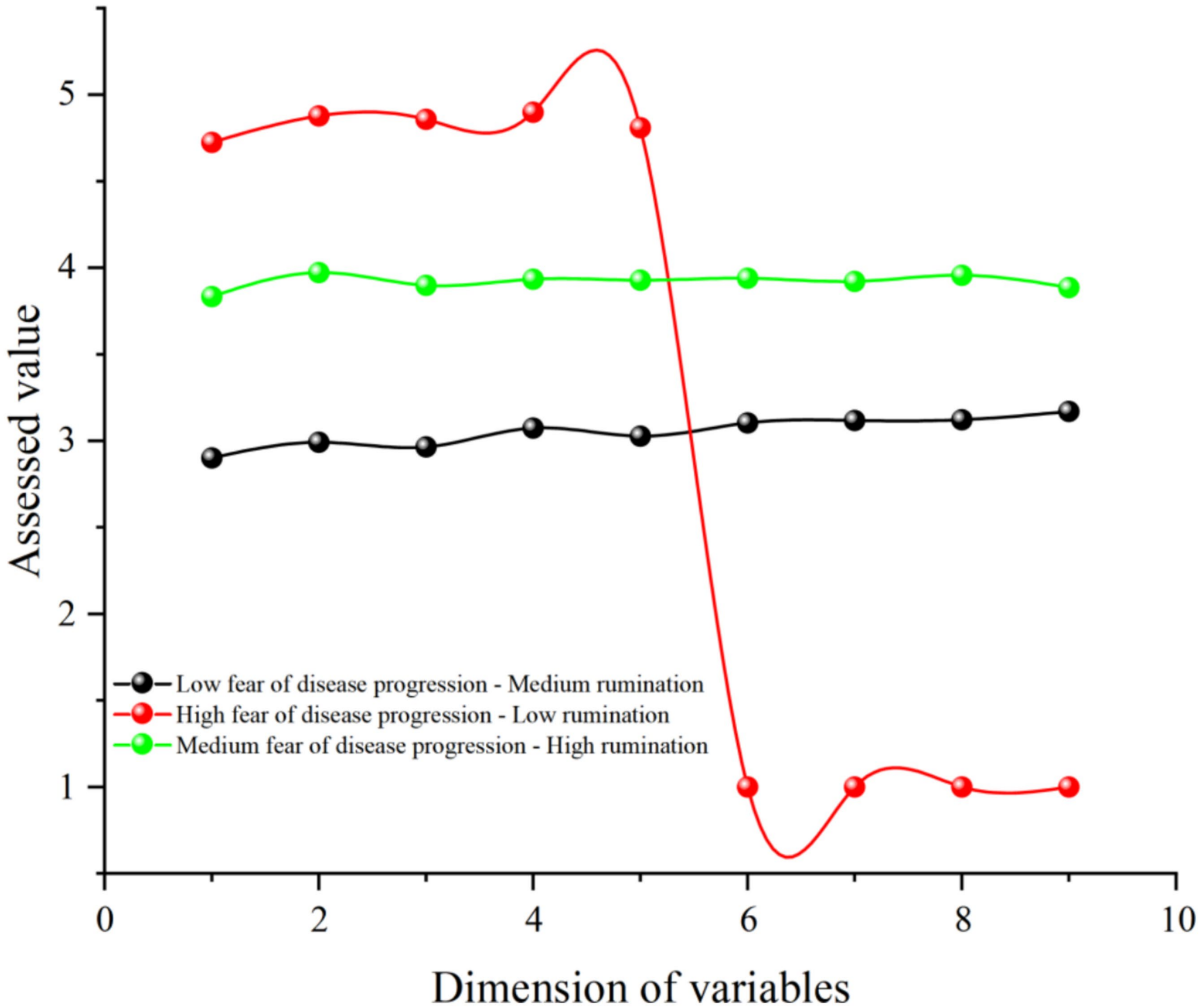
Potential subgroup profile of fear of disease progression and rumination in patients with melanoma. Dimensions 1–5 are fear of disease progression and dimensions 6–9 are rumination.

Based on the results of the 3-class latent profile analysis, we visualized the findings using Origin 2021 software, as shown in Figure 2. The first latent subgroup was characterized by low fear of disease progression and moderate rumination, accounting for 62.5%. The second latent subgroup was characterized by high fear of disease progression and low rumination, accounting for 1.4%. The third latent subgroup was characterized by moderate fear of disease progression and high rumination, accounting for 36.1%. This indicates that melanoma patients exhibit different response patterns concerning fear of disease progression and rumination.

One-way ANOVA of the latent subgroups and sleep disturbances, as shown in Table 7, revealed that the sleep disturbances of the moderate fear of disease progression-high rumination subgroup (*M* = 3.693, SD = 0.378) were significantly higher than those of the low fear of disease progression-moderate rumination subgroup (*M* = 3.159, SD = 0.448) and the high fear of disease progression-low rumination subgroup (*M* = 2.915, SD = 1.799). Therefore, the latent subgroups of fear of disease progression and rumination in melanoma patients significantly affect sleep disturbances [*F*(2, 498) = 78.187, *p* < 0.001, *η*^2^ = 0.239].

**Table 7:** Univariate analysis table of potential subgroups of fear of disease progression and rumination and sleep disturbance in patients with melanoma.

Potential subgroups	*M*	SD	*F*	*p*	*η* ^2^
Low fear of disease progression-medium rumination	3.159	0.448	78.187	<0.001	0.239
High fear of disease progression-low rumination	2.915	1.799			
Medium fear of disease progression-high rumination	3.693	0.378			

## 4 Discussion

### 4.1 Variable-centered analysis

This study revealed a significant positive association between fear of disease progression and sleep disturbances in melanoma patients through variable-centered analysis. This finding is highly consistent with existing literature, emphasizing the direct negative impact of cancer-related psychological fear on sleep health (58, 59). The results indicate that fear of disease progression directly association sleep disturbances, suggesting that patients’ persistent worries about tumor recurrence or metastasis significantly exacerbate symptoms such as difficulty falling asleep, nighttime awakenings, and early waking. This relationship can be traced back to physiological mechanisms: fear of disease progression activates the sympathetic nervous system, leading to hyperactivity of the hypothalamic–pituitary–adrenal (HPA) axis, which in turn disrupts sleep architecture (60, 61). For example, in melanoma patients, due to the high metastatic potential of the disease and treatment uncertainties, this fear often translates into heightened cognitive vigilance, where catastrophic interpretations of bodily signals further prolong sleep latency (62, 63). Melanoma patients experience a higher incidence of sleep disturbances, potentially due to their unique neurotoxic treatments such as chemotherapy and targeted therapies (64, 65).

This study confirms the mediating role of rumination between fear of disease progression and sleep disturbances, highlighting the hierarchical relationship—but not redundancy—between these two constructs. Conceptually, fear of disease progression represents a primary affective-cognitive appraisal of health threats, characterized by vigilance, worry, and uncertainty regarding tumor recurrence. Rumination, by contrast, constitutes a secondary meta-cognitive process that prolongs and amplifies this primary fear through repetitive self-focused thought and failure to disengage attention from threat cues (66). In other words, fear of disease progression captures the content of disease-related fears, whereas rumination reflects the process by which these fears are maintained and exacerbated over time (67).

Empirically, the discriminant validity tests corroborate that these constructs are statistically separable. Hence, while conceptually interrelated within a broader stress–appraisal–coping framework, fear of disease progression and rumination operate on distinct cognitive levels: the former denotes threat appraisal, and the latter perseverative elaboration, together forming a sequential psychological cascade contributing to sleep disturbance.

According to the Response Styles Theory (68), rumination amplifies negative emotions and depletes cognitive resources, making it difficult for patients to disengage from repetitive thoughts about disease threats, thus reinforcing cognitive arousal before sleep (69). Specifically, this study found that fear of disease progression significantly predicts rumination, which in turn exacerbates sleep disturbances. This pathway aligns with Attention Control Theory (41), which posits that rumination enhances attentional bias and memory retrieval, leading to catastrophic thinking about neutral events, ultimately inhibiting autonomic nervous system calming. The mediating pathway of rumination explains why patients with high fear are more likely to fall into a vicious cycle of sleep disturbances. Rumination not only maintains the intensity of fear but also promotes tumor microenvironment remodeling by upregulating inflammatory factors, indirectly exacerbating sleep problems.

### 4.2 Latent profile analysis

Using latent profile analysis, this study identified three heterogeneous subgroups of melanoma patients regarding fear of disease progression and rumination. This method addresses the limitations of variable-centered analysis, which assumes homogeneity within the group while neglecting individual differences. Specifically, the first subgroup (low fear of disease progression-moderate rumination, comprising 62.5%) represents the majority of patients, displaying lower fear levels and moderate rumination, likely reflecting better psychological adaptation mechanisms, such as effective coping strategies or supportive social networks. This subgroup exhibited relatively low levels of sleep disturbances. For instance, Tian and Wang (70) noted that melanoma patients with low fear of disease progression often maintain sleep stability through positive cognitive restructuring. The second subgroup (high fear of disease progression-low rumination, comprising 1.4%) shows intense fear but lower rumination, which may stem from individual traits like high resilience or external interventions (e.g., medication), preventing negative thoughts from being amplified. However, given its extremely small proportion, the findings related to this subgroup should be interpreted with great caution. Such a small class may reflect sample idiosyncrasy or unstable model estimation rather than a stable psychological pattern (71). Thus, these results are exploratory and cannot support firm conclusions about its psychological mechanisms or clinical significance. This subgroup had the lowest sleep disturbances, supporting the Response Styles Theory that low rumination can buffer the negative impact of fear on sleep (72). The third subgroup (moderate fear of disease progression-high rumination, comprising 36.1%) exhibited moderate fear of disease progression but the highest rumination, resulting in significantly higher sleep disturbances. This indicates that rumination acts as a reinforcing persistent effect of fear, leading to cognitive arousal (73). The subgroup classifications from the latent profile model not only validate Attention Control Theory, where rumination depletes cognitive resources leading to delayed sleep onset, but also provide a basis for personalized interventions. For example, cognitive-behavioral therapy targeting the high rumination subgroup could prioritize interrupting thought cycles. More importantly, by synthesizing variable-centered and person-centered approaches within a unified analytical framework, this study advances methodological innovation in psycho-oncology research. The variable-centered mediation model clarifies the general mechanism through which fear of disease progression influences sleep disturbance via rumination, whereas the person-centered analysis delineates heterogeneity across psychological profiles. Integrating these two perspectives provides a theoretically cohesive understanding that is both generalizable and individualized—addressing the dual need for mechanism identification and personalized care. Such integration embodies an emergent paradigm shift in behavioral medicine, emphasizing the transition from “average-based inference” toward “precision mental health mapping” tailored to disease-specific contexts.

Compared to traditional variable-centered approaches, latent profile analysis more effectively captures clustering patterns of psychological characteristics in melanoma patients. The three subgroup classifications highlighted the interactive heterogeneity of fear of disease progression and rumination. Detailed analysis showed that the low fear of disease progression-moderate rumination subgroup, as the dominant pattern, may benefit from early diagnosis or supportive care, reducing the occurrence of catastrophic thinking. Although the high fear of disease progression-low rumination subgroup represents the smallest proportion, its unique characteristics challenge linear assumptions. Intense fear of disease progression without high rumination suggests the role of protective factors such as intrinsic resilience or external resources. This subgroup exhibited lower sleep disturbances, corroborating (74), which found that low rumination blocks the transmission of fear to physiological symptoms. However, the prominent issues in the moderate fear of disease progression-high rumination subgroup reveal potential risks. Moderate fear of disease progression is amplified by high rumination, leading to cognitive biases and emotional persistence, resulting in significantly decreased sleep quality. This aligns with (75), which found that rumination predicts the persistence of anxiety, as high rumination patients may overinterpret bodily signals, leading to pre-sleep vigilance.

### 4.3 Practical implications

This study integrates variable-centered and person-centered analysis methods, revealing the critical mediating role of rumination in the relationship between fear of disease progression and sleep disturbances in melanoma patients, while identifying a psychologically high-risk subgroup characterized by high fear and high rumination. These findings have significant clinical implications. First, the study underscores the necessity of systematic psychological screening for melanoma patients. Current clinical practice often emphasizes the management of physiological symptoms while neglecting the assessment of psychological mechanisms. This study recommends routinely incorporating standardized assessment tools for fear of disease progression and rumination into oncology and dermatology outpatient settings to facilitate early identification of high-risk individuals. Particularly for patients in the mid-treatment or recurrence stage, regular monitoring of psychological status should be conducted to implement interventions before sleep disturbances develop into chronic issues. Additionally, healthcare providers should receive relevant training to enhance their sensitivity to patients’ psychological needs, allowing for the integration of psychological assessment results into overall treatment plans, achieving a holistic model of integrated care.

Secondly, this study provides empirical evidence for developing targeted psychological intervention measures. The current findings highlight the mediating role of rumination in the association between fear of disease progression and sleep disturbance, offering potential implications for psychological intervention. Importantly, these implications are evidence-informed, exploratory, and non-prescriptive, given the cross-sectional nature of the study. Cognitive-behavioral therapy (CBT) may represent a promising, evidence-informed approach to mitigating maladaptive rumination and alleviating fear-related distress. Techniques such as cognitive restructuring, mindfulness-based awareness, and emotional regulation could potentially help patients detach from repetitive negative thoughts that contribute to cognitive arousal and sleep disruption. These interpretations are theoretical hypotheses that require confirmation through future longitudinal and interventional research. However, because the current study is cross-sectional, these interpretations should be viewed as theoretical guidance rather than evidence of causal efficacy. Rumination-focused cognitive interventions and psychoeducational programs may also be explored as complementary approaches to support melanoma patients in managing anxiety and improving overall sleep hygiene. Yet, these approaches remain untested within melanoma-specific populations, and their practical efficacy awaits confirmation through rigorous longitudinal and experimental studies. Furthermore, considering the multidimensional nature of fear of disease progression (e.g., worries about family and occupational functioning), intervention plans should also include family support discussions and psychoeducational content to help patients and their families understand the disease process and reduce anxiety stemming from information uncertainty. In terms of implementation, in addition to traditional face-to-face consultations, digital intervention platforms could be developed to improve accessibility and adherence, especially suitable for areas with underdeveloped medical resources.

Finally, this study calls for establishing a multidisciplinary collaborative (MDT) psychological support network within the oncology care system. This includes the involvement of oncologists, nurses, psychotherapists, and social workers to form an integrated process of “assessment-intervention-follow-up.” For example, after a patient’s diagnosis, a nurse could conduct preliminary psychological screening, and identified high-risk cases could be referred to psychotherapists for further assessment and individualized intervention, while social workers could provide resource linkage to alleviate the impact of economic or social pressures on psychological status. Moreover, healthcare institutions should prioritize the long-term management of patients’ sleep quality, integrating sleep hygiene education into routine health guidance and combining non-pharmacological interventions (e.g., sleep restriction therapy, relaxation training) to improve sleep architecture. Through this systematic integrated practice, it can not only alleviate patients’ psychological distress and sleep disturbances but also indirectly enhance treatment adherence and improve immune function, thus positively impacting long-term quality of survival.

### 4.4 Cultural considerations

The psychological process observed in this study should also be interpreted within the Chinese cultural context, where collectivist values and family-centered norms strongly shape coping tendencies. Within collectivist societies, maintaining relational harmony often leads individuals to regulate emotional expression and rely on social connectedness rather than self-disclosure of distress (76). For Chinese melanoma patients, this cultural orientation may have dual effects on fear of disease progression and rumination. On one hand, family cohesion and interdependence can serve as protective resources, buffering fear through shared coping and emotional support (77). On the other hand, the tendency to suppress personal worries to avoid burdening family members may unintentionally foster persistent internal reflection and increase ruminative thinking (66).

Therefore, collectivist coping patterns might help explain why some patients maintain a high level of internalized fear and cognitive preoccupation even when social support is abundant. Future cross-cultural research could further investigate how shared cultural values moderate the pathways between fear, rumination, and sleep disturbance, thereby enriching culturally sensitive psycho-oncological care models.

### 4.5 Limitations and future research directions

Despite employing a dual-perspective analytical approach that elucidates the mediating role of rumination in the relationship between fear of disease progression and sleep disturbances in melanoma patients, several methodological limitations remain. First, the cross-sectional design of the study cannot establish causal relationships between variables. For instance, fear of disease progression may trigger rumination and subsequently exacerbate sleep disturbances, but conversely, sleep disturbances may amplify negative cognitions, thereby reinforcing fear and rumination. This temporal ambiguity limits the dynamic interpretation of the mechanisms involved. Future research should incorporate longitudinal designs, such as prospective cohort studies, to track temporal changes among variables and validate the mediating pathways more accurately.

Secondly, while the convenience sampling method is efficient, it may introduce selection bias. The study sample primarily comes from three tertiary hospitals in Sichuan Province, where patients are mostly urban residents with higher educational levels. This may overestimate the severity of psychological problems in the overall population, as rural or lower-educated patients may not have been included due to limited medical resources. Future studies should aim to include a more diverse geographical and socio-economic population to enhance external validity. Additionally, exploring potential moderating factors such as treatment stage, type of therapeutic intervention, and social support can help identify environmental factors influencing the observed relationships.

Finally, self-reported questionnaires rely on patients’ subjective perceptions. Although we ruled out significant common method bias through Harman’s single-factor test, responses may still be affected by social desirability bias or recall bias, particularly when assessing cognitive processes such as rumination. While the research scales have undergone cultural adaptation, the original scale tools (e.g., Fear of Disease Progression Scale) were developed in a Western context, which may have subtle biases in capturing culture-specific expressions (e.g., collectivist-oriented family concerns) of Chinese patients. Future research should develop culturally sensitive tools to enhance measurement sensitivity. Additionally, including objective indicators of sleep quality could provide more reliable and physiologically grounded data. Moreover, incorporating clinical assessments or behavioral indicators of fear and rumination will improve the structural validity of the research.

Although our mediation model visually depicts directional pathways, it is important to emphasize that these represent theoretical relationships. Owing to the cross-sectional nature of our data, the present findings indicate associational, not causal, links among fear of disease progression, rumination, and sleep disturbance. Any interpretations regarding directionality should therefore be treated as hypothesis-generating and subject to future longitudinal verification.

## 5 Conclusion

This study employs a dual-perspective analytical framework, elucidating the critical mediating role of rumination in the relationship between fear of disease progression and sleep disturbances in melanoma patients. Variable-centered analysis confirms that fear of disease progression not only directly exacerbates sleep disturbances but also indirectly amplifies sleep disturbances through increased rumination. Additionally, the person-centered approach identifies different psychological subgroups, revealing that individuals with heightened fear of disease progression and rumination constitute a high-risk subgroup experiencing the most severe sleep disturbances. Furthermore, interpretations regarding the smallest latent subgroup (1.4%) should be treated as preliminary, given its limited statistical stability and representativeness. These findings emphasize the necessity of integrating individualized psychological assessments and interventions into routine oncology treatment. Specifically, cognitive-behavioral and rumination-focused strategies may serve as promising, evidence-informed directions for psychological care in melanoma populations. Nevertheless, given the cross-sectional design of this study, these approaches should be considered exploratory recommendations, not empirically confirmed interventions. Future longitudinal and interventional research is needed to validate these causal pathways and explore personalized treatment approaches for different patients.

## Data Availability

The raw data supporting the conclusions of this article will be made available by the authors, without undue reservation.
